# How the forebrain transitions to adulthood: developmental plasticity markers in a long-lived rodent reveal region diversity and the uniqueness of adolescence

**DOI:** 10.3389/fnins.2024.1365737

**Published:** 2024-02-22

**Authors:** B. Maximiliano Garduño, Patrick Hanni, Chelsea Hays, Patricia Cogram, Nathan Insel, Xiangmin Xu

**Affiliations:** ^1^Department of Anatomy and Neurobiology, School of Medicine, University of California, Irvine, Irvine, CA, United States; ^2^Department of Psychology, University of Montana, Missoula, MT, United States; ^3^Department of Ecological Sciences, Faculty of Sciences, Institute of Ecology and Biodiversity, Universidad de Chile, Santiago, Chile; ^4^The Center for Neural Circuit Mapping, University of California, Irvine, Irvine, CA, United States; ^5^Department of Psychology, Wilfrid Laurier University, Waterloo, ON, Canada; ^6^Institute for Memory Impairments and Neurological Disorders, University of California, Irvine, Irvine, CA, United States

**Keywords:** plasticity, degu (*Octodon degus*), perineuronal net (PNN), microglia, adolescence

## Abstract

Maturation of the forebrain involves transitions from higher to lower levels of synaptic plasticity. The timecourse of these changes likely differs between regions, with the stabilization of some networks scaffolding the development of others. To gain better insight into neuroplasticity changes associated with maturation to adulthood, we examined the distribution of two molecular markers for developmental plasticity. We conducted the examination on male and female degus (*Octodon degus*), a rodent species with a relatively long developmental timecourse that offers a promising model for studying both development and age-related neuropathology. Immunofluorescent staining was used to measure perineuronal nets (PNNs), an extracellular matrix structure that emerges during the closure of critical plasticity periods, as well as microglia, resident immune cells that play a crucial role in synapse remodeling during development. PNNs (putatively restricting plasticity) were found to be higher in non-juvenile (>3 month) degus, while levels of microglia (putatively mediating plasticity) decreased across ages more gradually, and with varying timecourses between regions. Degus also showed notable variation in PNN levels between cortical layers and hippocampal subdivisions that have not been previously reported in other species. These results offer a glimpse into neuroplasticity changes occurring during degu maturation and highlight adolescence as a unique phase of neuroplasticity, in which PNNs have been established but microglia remain relatively high.

## 1 Introduction

During nervous system development, the establishment of local and long-range connections requires high levels of synaptic plasticity (Semple et al., 2013). As animals reach sexual maturity and adulthood, plasticity stabilizes in ways that permit adaptability without disrupting circuit function (Tau and Peterson, 2010). This transition to adulthood is not immediate, nor does it follow the same timecourse across different brain regions (Dumontheil et al., 2008; Shaw et al., 2008; Reh et al., 2020). Sitting between this transition from juvenile to adult life phases is adolescence, an intermediate developmental period that may offer a unique window into neuroplasticity in which many essential circuits have been established but the brain is still highly adaptable (Fuhrmann et al., 2015; Larsen and Luna, 2018). Previous non-human primate studies found there is rapid synaptic density increases during early life stages that produce an overabundance of synapses that persist through adolescence at levels higher than in adulthood (Rakic et al., 1994). Other studies identified that childhood and adolescence are implicated in the onset of neuropsychiatric conditions, and that many of these conditions can be traced to developmental processes, including dysfunctional plasticity (Kessler et al., 2007; Citri and Malenka, 2008; Insel, 2010; Semple et al., 2013; Solmi et al., 2022; Appelbaum et al., 2023). Unfortunately, we still lack fundamental knowledge on the timecourse of these processes, and how they map onto maturation phases across brain regions.

Perineuronal nets (PNNs) are specialized extracellular matrix (ECM) structures involved in synaptic plasticity and memory modulation (Tsien, 2013; Sorg et al., 2016; Reichelt et al., 2019; Carulli and Verhaagen, 2021). Emergence of these structures coincides with the closure of plasticity-rich critical periods (Pizzorusso et al., 2002; Hensch, 2005; Carulli and Verhaagen, 2021), thought to be due to PNN's ability to stabilize synapses and trigger circuit maturation (Pizzorusso et al., 2002; Fawcett et al., 2019; Venturino et al., 2021). PNNs therefore appear to prevent circuit changes once they have been appropriately tuned by early experience. In contrast to PNNs, which restrict plasticity, microglia mediate certain forms of developmental plasticity (Wu et al., 2015; Cornell et al., 2022). As the brain's resident immune cell, microglia can remove cells and remodel the ECM and synapse architecture. They prune excessive synapses during early brain development and continue regulating synapse dynamics in adulthood. Together, markers associated with microglia (i.e., ionized calcium binding adaptor molecule 1, Iba1) and PNNs (i.e., wisteria floribunda lectin, WFA) offer complimentary indices into ECM and glial plasticity changes occurring during brain maturation.

Here we examine distributions of PNNs and microglia in the degu (*Octodon degus*), a highly social caviomorph rodent endemic to Chile. Degus are born precocious (i.e., with open eyes and able to move independently) and reach sexual maturity around 3.5 months of age, offering a developmental window to study experience-dependent plasticity that is longer than that of mice and rats (Hummer et al., 2007; Mahoney et al., 2011; Ardiles et al., 2013). Degus reach adulthood at around 1 year of age and can live up to 5–8 years in captivity, considerably longer than the 2-year lifespan of mice and rats (Ebensperger, 2001; Tan et al., 2022). Most degus do not reach 2 years of age in the wild, and previous studies identified species-typical behavior deficits in 3-year-old degus, suggesting the onset of an aging processes in the degu brain (Ardiles et al., 2013; Deacon et al., 2015). Degus have already been used to investigate developmental plasticity (Akers et al., 2014), social development (Wilson, 1982; Ovtscharoff and Braun, 2001; Colonnello et al., 2011; Malcangi et al., 2020), and have also received attention in studies of diurnal circadian rhythms (Hagenauer and Lee, 2008; Bauer et al., 2019), vision (Jacobs et al., 2003), Alzheimer's disease (Hurley et al., 2022; Tan et al., 2022), and social behavior (Long, 2007; Quirici et al., 2008; Insel et al., 2020; Rivera et al., 2020; Lidhar et al., 2021). We characterize the neurodevelopmental patterns of PNN and microglia expression across several divisions of the degu forebrain. Our underlying interest in lifespan memory focuses our investigation on the hippocampal system, including hippocampal subregions, cortical regions connected with the hippocampus (prelimbic, entorhinal cortex, and retrosplenial cortex), the amygdala, and the nucleus reunions of the thalamus. These regions, in addition to primary somatosensory cortex, provide a sampling from different structures, cortical lobes, hierarchical levels, and subcircuits. A total of 16 brain regions from 1 to 40 month old degus were examined to identify commonalities and differences between them, as well as evident timecourse differences between levels of PNN and microglia.

## 2 Materials and methods

### 2.1 Experimental design and statistical analyses

Degus (*O. degus*) used in this study came from two sources (1) a breeding colony at the University of Montana (*n* = 28) and (2) a colony stocked using outbred (pups from pregnant females caught from the wild) animals at the Institute of Ecology and Biodiversity, University of Chile, Santiago, Chile (*n* = 4). No apparent differences were observed between degus from the two sources, and they were thus pooled together. All degus were handled and euthanized in accordance with protocols approved by ethics and Institutional Animal Care and Use Committees as part of retirement from breeding and experimental protocols (AUPs 036-18NIPSYC-061918, 033-18NIPSYC-060618, and 001-19NIPSYC-031919). Only degus without prior exposure to pharmacological or invasive procedures were used, and no animals had been subject to significant social or other stressors. All degus were aged in captivity since birth, kept on a 12:12 h light/dark cycle, housed in groups of 2–4, provided with enrichment (chew blocks, nylon bones, shelters, regular dust baths), and given *ad libitum* water and food.

The age of the 32 degus (13 males, 19 females) used in the study ranged from 1 to 40 months. Animals were assigned to 4 age groups as follows: 8 juveniles (1–3 months old), 11 adolescents (5–8 months old), 7 younger adults (12–19 months old), and 6 older adults (23–40 months old; Table 1). The age choice for juvenile and adolescent groups was based on observations of puberty onset within the colony (appearance of penile spikes and vaginal openings) as well as prior research suggesting that adolescence typically begins around 3–4 months of age (Hummer et al., 2007; Mahoney et al., 2011; Suckow et al., 2012).

**Table 1:** Individual degu information.

**ID**	**Age (months)**	**Sex**	**Classification**
180307	1	Male	Juvenile (1–3 m.o.)
140202	2	Male	Juvenile (1–3 m.o.)
140203	2	Male	Juvenile (1–3 m.o.)
150404	2	Male	Juvenile (1–3 m.o.)
6030	3	Female	Juvenile (1–3 m.o.)
6033	3	Male	Juvenile (1–3 m.o.)
6039	3	Female	Juvenile (1–3 m.o.)
6050	3	Male	Juvenile (1–3 m.o.)
120101	5	Male	Adolescent (5–8 m.o.)
120102	5	Male	Adolescent (5–8 m.o.)
140103	5	Female	Adolescent (5–8 m.o.)
140104	5	Female	Adolescent (5–8 m.o.)
140401	6	Female	Adolescent (5–8 m.o.)
140402	6	Female	Adolescent (5–8 m.o.)
150301	6	Female	Adolescent (5–8 m.o.)
160203	8	Female	Adolescent (5–8 m.o.)
160204	8	Female	Adolescent (5–8 m.o.)
160205	8	Female	Adolescent (5–8 m.o.)
160206	8	Female	Adolescent (5–8 m.o.)
050301	12	Male	Younger adult (12–19 m.o.)
060501	12	Female	Younger adult (12–19 m.o.)
090202	13	Female	Younger adult (12–19 m.o.)
120105	13	Female	Younger adult (12–19 m.o.)
060403	16	Female	Younger adult (12–19 m.o.)
060302	19	Female	Younger adult (12–19 m.o.)
060303	19	Female	Younger adult (12–19 m.o.)
060505	23	Male	Older adult (23–40 m.o.)
060406	27	Male	Older adult (23–40 m.o.)
010306	32	Male	Older adult (23–40 m.o.)
04604	36	Male	Older adult (23–40 m.o.)
010503	36	Female	Older adult (23–40 m.o.)
020103	40	Female	Older adult (23–40 m.o.)

Detailed information of the 32 degus used in the study, including ID number, age, sex, and age group classification (8 juveniles, 11 adolescents, 7 younger adults, and 6 older adults).

An exclusion criterion was applied to ensure tissues with very poor staining were not included in data analysis. This criterion identified outliers as brain regions where both PNN (WFA) and Iba relative intensity levels were two standard deviations from the corresponding degu's age group mean. Only one data point fit this criteria, a 19-month-old degu's prelimbic cortex, where both values were close to zero, indicating poor staining in that brain slice. This single point exclusion did not have considerable effects on linear regression analyses (slightly smaller *p*-values, although both PNN and Iba1 were already significant with the outlier) but did reveal a significant PNN difference between juvenile and younger adult degu prelimbic cortex. Iba1 relative intensity remained without any significant age group differences after outlier removal.

Statistical analyses were performed using GraphPad Prism 9 (GraphPad Software, CA, USA) and custom python scripts. Simple linear regression plots display *R*^2^ values, corresponding *p*-values, and show 95% confidence bands flanking the top and bottom of the linear regression line. Comparisons between degu age groups were conducted using Kruskal–Wallis nonparametric one-way ANOVA tests and were followed by *post-hoc* Dunn's tests of all head-to-head age group comparison permutations: ^trend^*p* < 0.1, ^*^p < 0.05, ^**^p < 0.01, ^***^p < 0.001, ^****^p < 0.0001.

Sigmoid inflection analysis was performed using the logistic sigmoid function:


$$\begin{array}{l} y=\frac{L}{1+e^{-k\left(x-x_{0}\right)}} \end{array}$$

Data were fitted using a least squares regression method with 1,000 iterations. Constraints were placed on specific parameters (|*k*| > 0.5; *x*_0_∈ [1, 40]; *L unconstrained*) to ensure sigmoid plot inflection points lay between the ages of the degus studied (1 to 40 months). An improved model with additional constrains (|*k*|> 0.5; *x*_0_ ∈ [1, 40]; *L* ≤ max(*PNN*/*mm*^2^ *or Iba1 relative intensity*)); *b* ≤ min(*PNN*/*mm*^2^ *or Iba1 relative intensity*) was used to obtain a better fit.


$$\begin{array}{l} y=\frac{L}{1+e^{-k\left(x-x_{0}\right)}}+b \end{array}$$

Principal component analysis (PCA) was conducted using the sklearn.decomposition. PCA package. As not all degus had all anatomical ROIs available, the following regions were included in PCA analysis: *PNN—*RSC, TRN, CA3a, S1, SUB; *Iba1*—RSC, TRN, CA3a, S1, CA1. The top two principal components (i.e., greatest eigenvalues) were used to plot PCA data. Centroids were calculated for each age group in PCA space, and Euclidean distances between each data point and age group centroids were calculated to assess intra- and inter-group distance differences. Multivariate analysis of variance (MANOVA) analysis was carried out on principal components 1 and 2 and followed by *post-hoc* univariate analysis of variance (ANOVA) and Tukey's HSD (honestly significant difference) tests.

### 2.2 Tissue preparation

Degus were euthanized by overdose of sodium pentobarbital (390 mg) and sodium phenytoin (50 mg; Euthansol) followed either by decapitation or cardiac perfusion. Right hemispheres were fixed in 4% paraformaldehyde (PFA) in 1 × phosphate buffer saline (PBS, pH 7.4) for 24 h at 4°C, then soaked in 30% sucrose in PBS for 3 days prior to sectioning. Brain coronal sections were cut at a 30 μm thickness using a Leica SM2010R or a Leica SM2000R sliding microtome. Serial free-floating coronal sections were harvested in 1 × PBS and transferred to cryoprotective solution for −20°C long term storage.

### 2.3 Immunofluorescence staining

Free-floating coronal sections were rinsed three times with 1 × phosphate buffered saline (PBS) prior to incubation in 5% normal donkey serum (NDS) blocking buffer (Jackson ImmunoResearch Laboratories, West Grove, PA; #017-000-121) containing 0.075% (v/v) Triton X-100 in 1 × PBS for 2 h. Sections were then incubated with primary antibodies and stained against perineuronal nets and microglia. Slices containing thalamic reticular nucleus were also stained for parvalbumin to facilitate its identification. Biotinylated Wisteria floribunda lectin/agglutinin (WFA, 1:1,000) was used to stain perineuronal nets. This lectin binds to a sulfation motif in the chondroitin sulfate glycosaminoglycan chains found in most PNNs (Fawcett et al., 2019). Microglia were identified using ionized calcium binding adaptor molecule 1 (Iba1), a well-established marker present in most microglial subtypes (Shapiro et al., 2009; Stratoulias et al., 2019). WFA and primary antibodies against parvalbumin (PV, 1:1,000) and microglia (Iba1, 1:1,000) were diluted in 5% NDS using proper dilutions (Table 2) for 72 h at 4°C. Sections were then washed three times in 1 × PBS and incubated with appropriate fluorescent secondary antibodies (1:200) against primary antibodies and Alexa Fluor 488-conjugated streptavidin (1:500) diluted in 1 × PBS (as detailed in Table 2) for 2 h at room temperature. Sections were washed three more times in 1 × PBS and incubated in 1 × PBS containing 4′,6-diamidino2phenylindole (DAPI; ThermoFisher; D1306, 10 μM) for 30 min at room temperature. Finally, sections were mounted and coverslipped with Fluoromount-G (SouthernBiotech, Birmingham, AL; #0100-01) for microscopic imaging.

**Table 2:** Reagents and resources with Research Resource Identifiers (RRID) tags.

**Reagent or resource**	**Source**	**Identifier**	**City and state**	**RRID**	**Dilution**
**Antibodies**					
Anti-Iba1 (rabbit polyclonal)	FUJIFILM WAKO	019-19741	Richmond, VA, USA	*AB_839504*	1:1,000
Anti-Parvalbumin (goat polyclonal)	Swant	PVG-213	Burgdorf, CHE	*AB_2650496*	1:1,000
Cy5 Donkey anti rabbit (secondary, polyclonal)	Jackson immuno research	711-175-152	West Grove, PA, USA	*AB_2340607*	1:200
Cy3 Donkey anti goat (secondary, polyclonal)	Jackson immuno research	705-165-147	West Grove, PA, USA	*AB_2307351*	1:200
**Stains**					
Wisteria Floribunda Lectin/Agglutinin (WFA, WFL), biotinylated	Vector laboratories	B-1355-2	Newark, CA, USA		1:1,000
Alexa Fluor 488 Streptavidin	Jackson Immuno Research	016-540-084	West Grove, PA, USA		1:500

Detailed information on antibodies and stains used in the study, including source, identifier/catalog number, RRID, and dilution used in immunofluorescent experiments.

### 2.4 Microscopy

Coronal hemisphere slice overviews were imaged using a high-throughput Olympus VS120 scanning system. Confocal microscopy was conducted using an Olympus FV3000 microscope to obtain high resolution images. Serial optical sections were captured for each slice using a 10 × or 20 × objective, with 1 × -3.94 × zoom using a 1.5 μm step interval for 12–15 slices (z-stack). The maximum intensity projection 2D images were processed using Olympus FV31S-SW Fluoview software (Version 2.6, Olympus Life Science). Identical imaging conditions were maintained for each anatomical brain region.

### 2.5 Image analysis

#### 2.5.1 Perineuronal nets (WFA)

The degu brain atlas (Kumazawa-Manita et al., 2018) was superimposed on epiflourescent scans of degu coronal hemispheres to delineate anatomical regions of interest (ROI). ROIs were then imported into ImageJ (FIJI) software for analysis and quantification. A threshold was used to remove background signal which was followed by particle analysis to determine true PNN signal areas (mm^2^). This area was divided by the typical size of a neuron-enclosing PNN to yield the total amount of PNNs, which was divided by the total ROI area to yield PNN/mm^2^.

#### 2.5.2 Microglia (Iba1)

Maximum intensity projection of confocal micrographs were produced using Olympus FV31S-SW Fluoview software (Version 2.6, Olympus Life Science). Relative intensity for Iba1 fluorescence was calculated by measuring mean intensity values and subtracting background signal from each image.

## 3 Results

### 3.1 General PNN and microglia expression patterns in the degu brain

**Figure 1:**
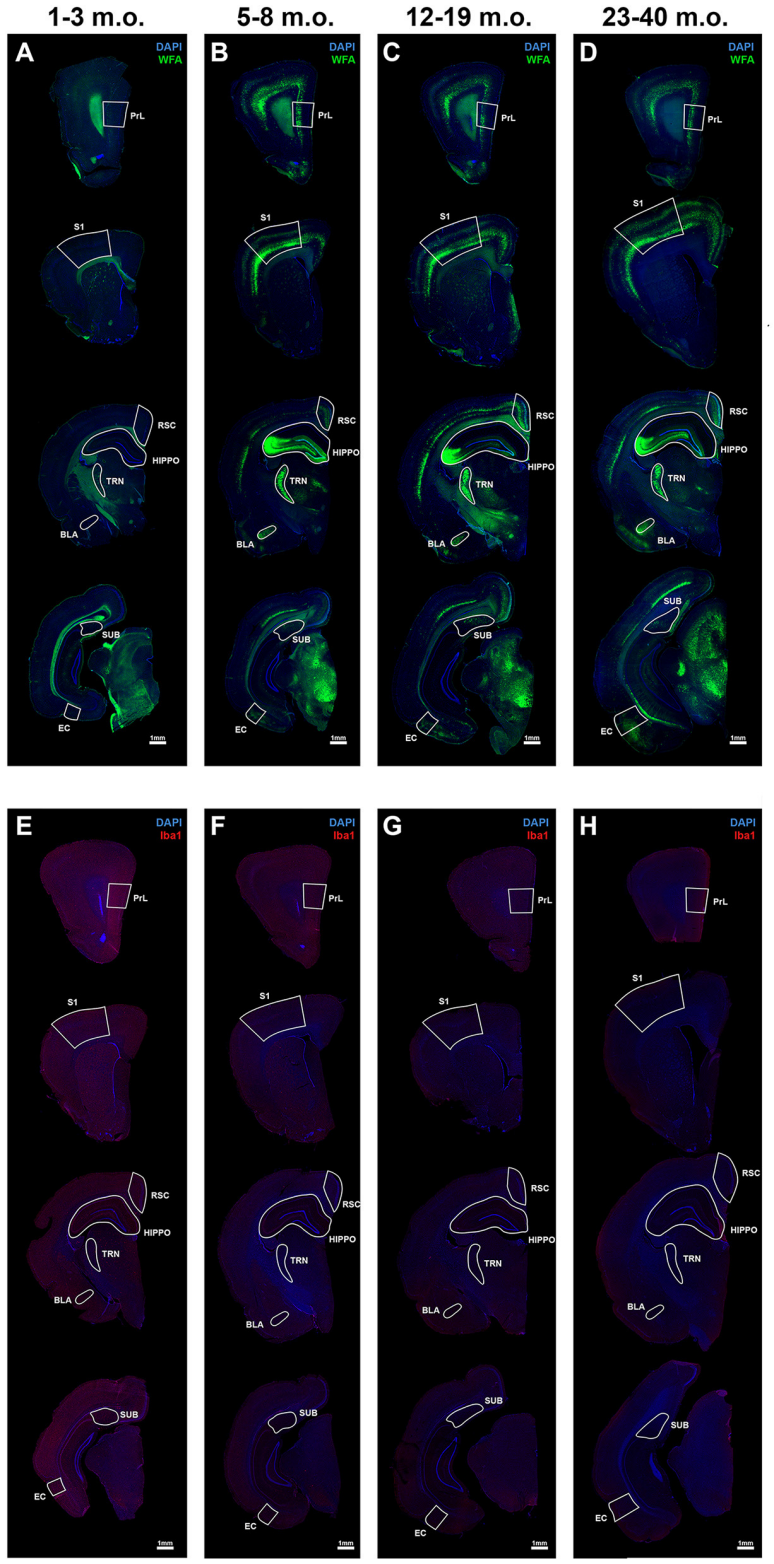
Perineuronal nets (PNNs) and microglia across the postnatal degu brain. **(A–H)** Immunofluorescence microscopic overviews of anterior-to-posterior coronal hemispheres of 1–3 m.o. (juvenile) **(A, E)**, 5–8 m.o. (adolescent) **(B, F)**, 12–19 m.o. (younger adult) **(C, G)**, and 23–40 m.o. (older adult) **(D, H)** degus. WFA stained PNNs (in green) showed increased signal across the post-puberty 5–40 m.o. degu brain **(B–D)** when compared to 1–3 m.o. degu **(A)**. Microglia immunoreactivity stained with Iba1 antibody (in red) showed greater signal in 1–3 m.o. degus **(E)** than older 5–40 m.o. degus **(F–H)**. Regions further investigated in this study are outlined and labeled. All sections were counterstained with DAPI (blue).

Expression of PNNs and microglia varied in an age and brain region dependent manner. Epiflourescent overviews showed PNNs are present in specific cortical and subcortical regions that span the rostrocaudal axis of the degu brain (Figures 1A–D). Further, these general PNN patterns were missing or less pronounced in juvenile degus (Figure 1A) and develop into their mature appearance with age. Microglia, on the other hand, showed an inverse pattern where juvenile degus exhibit enhanced immunoreactivity (Figure 1E) that decreased with age (Figures 1F–H), similar to what is seen in mice and humans (Brust et al., 2015; Lenz and Nelson, 2018; Menassa et al., 2022). To quantify these results, we focused on a selection of individual brain regions and then considered their common patterns and diversity.

### 3.2 Cortical regions in the juvenile degu exhibit low perineuronal net and high microglia expression patterns that invert with age

Four cortical regions were selected that offered a sampling across cortical lobes and hierarchy: the prelimbic cortex, entorhinal cortex, retrosplenial cortex, and primary somatosensory cortex.

**Figure 2:**
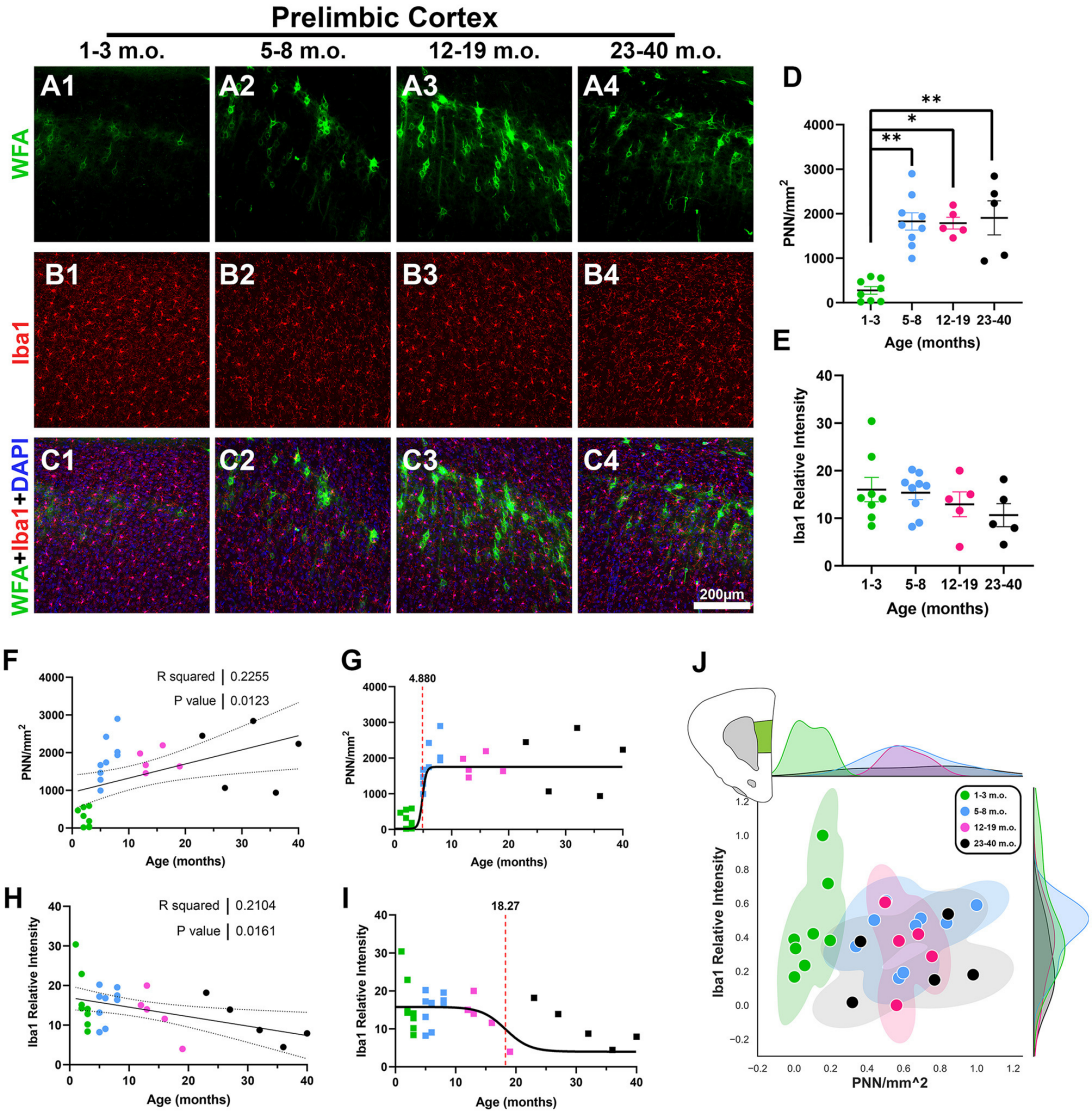
Prelimbic cortex (PrL) exhibits reduced perineuronal nets (PNN) in juvenile degus and an overall gradual decrease in microglia with age. **(A–C)** Immunofluorescence confocal micrographs showing PNNs (WFA, green) and microglia (Iba1, red) in the PrL of 1–3 m.o. (juvenile) **(A1, B1, C1)**, 5–8 m.o. (adolescent) **(A2, B2, C2)**, 12–19 m.o. (younger adult) **(A3, B3, C3)**, and 23–40 m.o. (older adult) **(A4, B4, C4)** degus. **(D)** Quantification of PNN-positive signal per mm^2^ in the four different age groups showed a significant difference between 1–3 m.o. degus and 5–8 m.o./12–19 m.o./23–40 m.o. degus (*p* = 0.003, *p* = 0.02, and *p* = 0.009, respectively). **(E)** Degu PrL had no significant Iba1 relative intensity differences between age groups. **(F, H)** Linear regression analysis of PNN/mm^2^ **(F)** and Iba1 relative intensity **(H)** with increasing degu age. PNN/mm^2^ possessed a significant positive correlation with age (*R*^2^ = 0.22; *p* = 0.012), while Iba1 relative intensity had a significant negative correlation (*R*^2^ = 0.21; *p* = 0.016). **(G, I)** Sigmoid curve data fits revealed PNNs have an earlier inflection age (4.88 months) than microglia (18.27 months) in the PrL. **(J)** Normalized individual data points for all 1–40 m.o. degus in PNN/mm^2^ and Iba1 relative intensity space showed juvenile degus separate from post-puberty age group clusters. Error bars represent SEM; Kruskal–Wallis test followed by *post-hoc* Dunn's test: ^trend^*p* < 0.1, *p < 0.05, **p < 0.01.

The prelimbic cortex (PrL), part of the rostral cingulate cortex (the medial prefrontal cortex), has been extensively studied for its role in context-dependent expectation and behavior (Granon and Poucet, 2000; Corbit and Balleine, 2003; Euston et al., 2012; Riaz et al., 2019; Green and Bouton, 2021; Kolk and Rakic, 2022). As seen in the confocal micrographs of Figures 2A–C, there was a clear jump from weak PNN staining in the juvenile degu to numerous PNNs in the adolescent, younger adult, and older adult degus. PNN density was significantly different between juvenile and adolescent/adult degus [Figure 2D; *H*_(3, 23)_ = 16.37, *p* = 0.001, Kruskal–Wallis (KW) ANOVA; *p* = 0.003, *p* = 0.02, *p* = 0.009, Dunn's test], and linear regression showed a significant increase with age (Figure 2F; *R*^2^ = 0.22, *p* = 0.012). Although microglia levels did not show significant differences between age groups (Figure 2E), there was a statistically significant negative linear regression over age (Figure 2H; *R*^2^ = 0.21 *p* = 0.016). We further characterized these datasets by fitting sigmoid curves to them to assess the temporal dynamics of these plasticity markers. PNNs showed a considerably younger inflection age (4.8 months) than microglia (18.2 months; Figures 2G, I). Relative levels of PNN and microglia are illustrated in Figure 2J.

**Figure 3:**
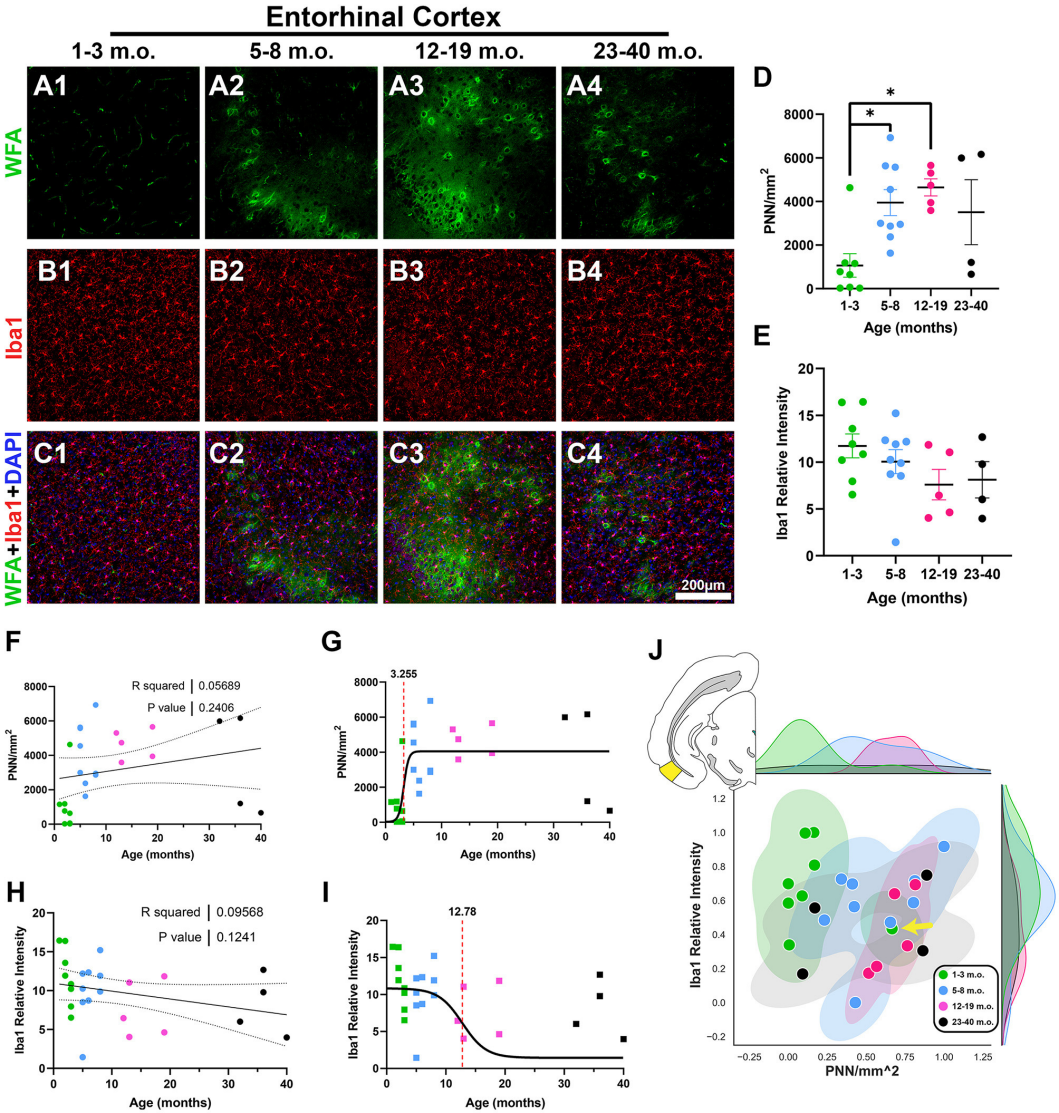
Juvenile degus show decreased perineuronal nets (PNN) in entorhinal cortex (EC) when compared to older degus. **(A–C)** Immunofluorescence confocal micrographs showing PNNs (WFA, green) and microglia (Iba1, red) in the EC of 1–3 m.o. (juvenile) **(A1, B1, C1)**, 5–8 m.o. (adolescent) **(A2, B2, C2)**, 12–19 m.o. (younger adult) **(A3, B3, C3)**, and 23–40 m.o. (older adult) **(A4, B4, C4)** degus. **(D)** Quantification of PNN-positive signal per mm^2^ in the four different age groups revealed significant differences between 1–3 m.o. degus and 5–8 m.o./12–19 m.o. degus (*p* = 0.039 and *p* = 0.025, respectively). **(E)** Microglia signal measured by Iba1 relative intensity showed no significant age group differences. **(F, H)** Linear regression analysis for PNN/mm^2^ **(F)** and Iba1 relative intensity **(H)** showed no significant correlation with age (*R*^2^ = 0.056, *p* = 0.24; *R*^2^ = 0.095, *p* = 0.124, respectively). **(G, I)** PNN exhibited an earlier inflection age (3.25 months) than microglia (12.78 months) in degu EC. **(J)** Normalized PNN/mm^2^ and Iba1 relative intensity 2D space highlighted a juvenile degu (yellow arrow) exhibiting adult-like PNN levels. Error bars represent SEM; Kruskal–Wallis test followed by *post-hoc* Dunn's test: ^trend^*p* < 0.1, *p < 0.05.

The entorhinal cortex (EC) is reciprocally connected with the PrL and plays an instrumental role in supporting temporal-spatial context for memory and cognition (Coutureau and di Scala, 2009; Takehara-Nishiuchi, 2014; Marks et al., 2021). PNNs were primarily present in layer 2–4 of the degu EC, but relatively absent in juvenile degus (Figures 3A–C). EC PNNs significantly increased with age, with juvenile degus having significantly lower PNN levels than adolescent and younger adult degus [*H*_(3, 22)_ = 11.15, *p* = 0.011, KW ANOVA; *p* = 0.039, *p* = 0.025, Dunn's test; Figure 3D]. No significant age effects were detected for levels of microglia (Figures 3E, H). Similar to PrL, the PNN inflection age (3.2 months) was substantially younger than in microglia (12.7 months; Figures 3G, I). One juvenile degu (yellow arrow in Figure 3J) exhibited robust PNN signal and lower Iba1 intensity, possibly indicating early EC PNN maturation in that degu.

**Figure 4:**
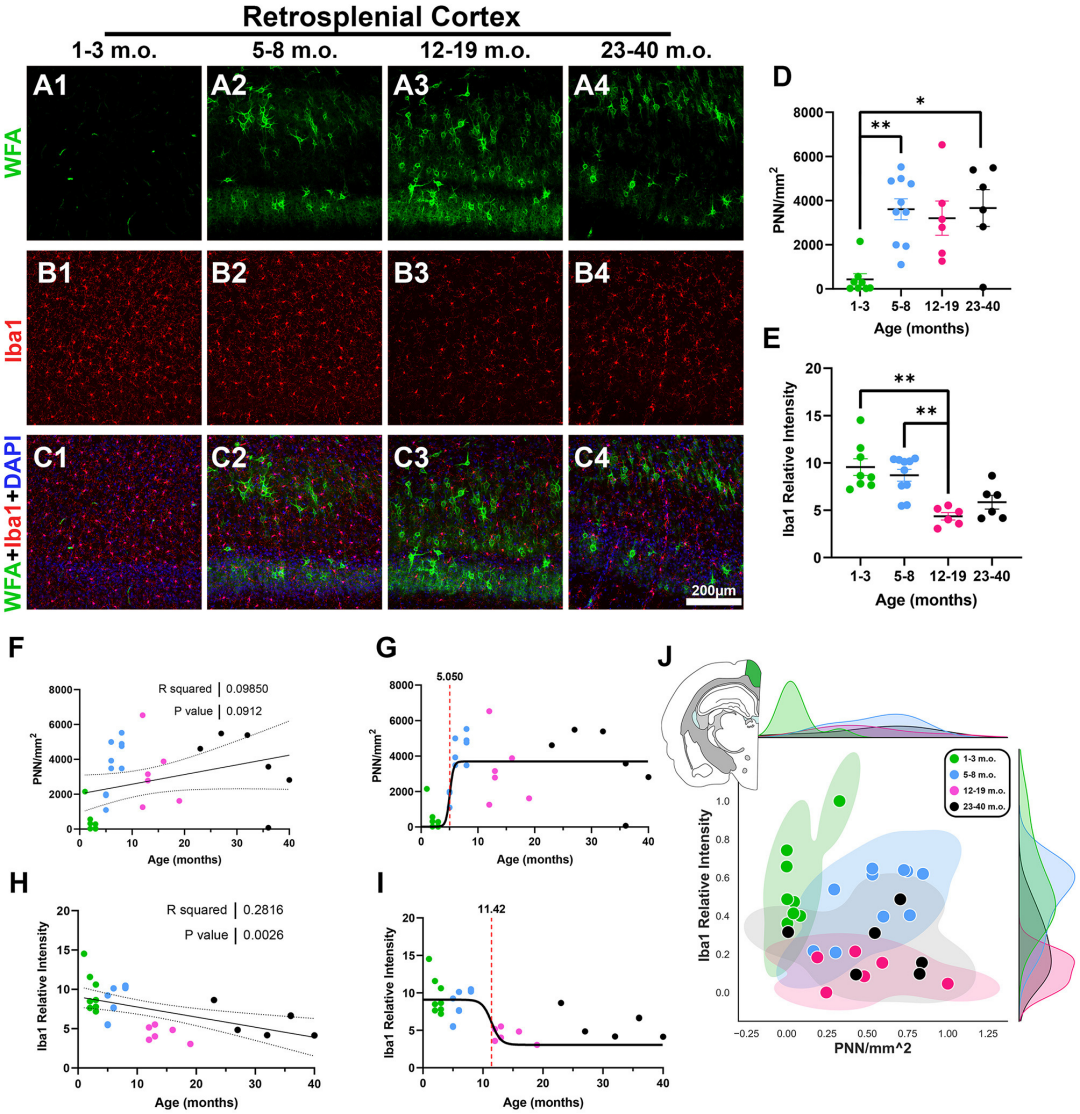
Degu retrosplenial cortex (RSC) exhibits decreasing levels of microglia with age while perineuronal nets (PNNs) are significantly decreased in juvenile degus compared to older counterparts. **(A–C)** Immunofluorescence confocal micrographs showing PNNs (WFA, green) and microglia (Iba1, red) in the RSC of 1–3 m.o. (juvenile) **(A1, B1, C1)**, 5–8 m.o. (adolescent) **(A2, B2, C2)**, 12–19 m.o. (younger adult) **(A3, B3, C3)**, and 23–40 m.o. (older adult) **(A4, B4, C4)** degus. **(D)** PNN density quantification showed a significant difference between 1–3 m.o. degus and 5–8 m.o./23–40 m.o. degus (*p* = 0.003 and *p* = 0.01, respectively). **(E)** 12–19 m.o. degus showed significantly less microglia signal than 1–3 m.o. and 5–8 m.o. degu RSC (*p* = 0.002, and *p* = 0.006, respectively). **(F, H)** Linear regression analysis of PNN densities with age **(F)** reveals a statistically trending positive correlation (*R*^2^ = 0.098; *p* = 0.091), while Iba1 relative intensity **(H)** exhibited a significant negative correlation (*R*^2^ = 0.281; *p* = 0.002). **(G, I)** PNNs possess an earlier sigmoid inflection age (5.05 months) than microglia (11.42 months) in the degu RSC. **(J)** Normalized PNN and Iba1 relative intensity 2D space showed substantial intermixing between post-puberty (5–40 m.o.) degu age groups. Error bars represent SEM; Kruskal–Wallis test followed by *post-hoc* Dunn's test: ^trend^*p* < 0.1, *p < 0.05, **p < 0.01.

The third cortical region analyzed was the retrosplenial cortex (RSC), a posterior complement to the rostral cingulate that is also connected with entorhinal and hippocampal memory systems and has important roles in learning and navigation (Vann et al., 2009). Juvenile degus exhibited very small levels of PNNs in their RSC while at the same time expressing strong Iba1 microglia signal (Figures 4A–C). Linear regression showed a significant Iba1 decrease with age (*R*^2^ = 0.28, *p* = 0.002), while PNN densities had an increasing statistical trend (*R*^2^ = 0.09, *p* = 0.09; Figures 4F, H). In line with this, juvenile degu RSC had significantly less PNN levels than adolescent and older adult degus [*H*_(3, 26)_ = 14.55, *p* = 0.002, KW ANOVA; *p* = 0.003, *p* = 0.01, Dunn's test; Figure 4D]. Younger adults exhibited significantly lower Iba1 relative intensities than their juvenile and adolescent counterparts [*H*_(3, 26)_ = 17.43, *p* = 0.0006, KW ANOVA; *p* = 0.002, *p* = 0.006, Dunn's test; Figure 4E]. Inflection ages were earlier for PNN (5.0 months) than microglia (11.4 months), indicative of earlier PNN maturation in the degu RSC (Figures 4G, I). When considering both PNN and microglia together, juvenile, adolescent, and younger adult groups appear to distribute across different vectors (Figure 4J).

**Figure 5:**
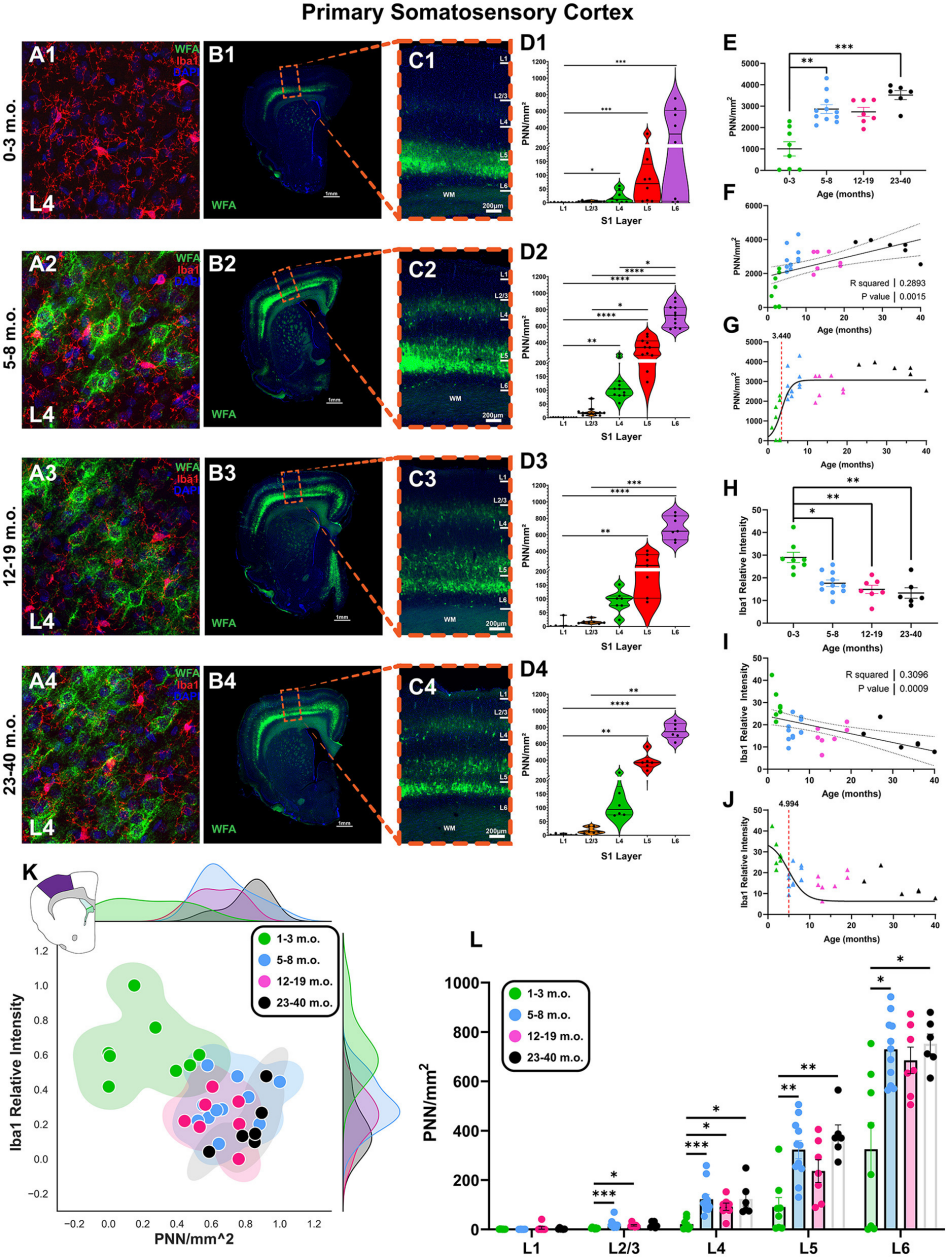
The degu primary somatosensory cortex (S1) exhibits a unique pattern of laminar perineuronal net (PNN) expression and is most plastic during pre-pubescence. **(A)** Confocal micrographs show PNNs (WFA, green) and microglia (Iba1, red) in S1's layer 4 from 1–3 m.o. (juvenile) **(A1)**, 5–8 m.o. (adolescent) **(A2)**, 12–19 m.o. (younger adult) **(A3)**, and 23–40 m.o. (older adult) **(A4)** degus. **(B)** Representative immunofluorescent overviews of S1-containing coronal hemispheres in 1–3 m.o. **(B1)**, 5–8 m.o. **(B2)**, 12–19 m.o. **(B3)**, and 23–40 m.o. **(B4)** degus. **(C)** Confocal micrograph zoom-in views from boxed areas in **(B)** depicting S1 cortical layers (PNN in green, DAPI in blue). **(D)** S1 layer-specific PNN/mm^2^ quantification for each degu age group. PNN densities were most elevated in deep S1 layers. **(E)** PNN quantification across all layers identified a significant difference between 1–3 m.o. degus and 5–8 m.o./23–40 m.o. degus (*p* = 0.004 and *p* = 0.0002, respectively). **(F)** PNN linear regression with age showed a significant positive correlation (*R*^2^ = 0.257; *p* = 0.003). **(H)** 1–3 m.o. degus possess greater microglia levels than 5–8 m.o., 12–19 m.o., and 23–40 m.o. degu S1 (*p* = 0.024, *p* = 0.005, and *p* = 0.001 respectively). **(I)** Iba1 relative intensity showed a significant negative correlation with age (*R*^2^ = 0.309; *p* = 0.0009). **(G, J)** Sigmoid data curve fittings revealed PNNs have an earlier inflection age (3.44 months) than microglia (4.99 months) in degu S1. **(K)** 2D plots of normalized S1 PNN/mm^2^ and microglia Iba1 levels showed substantial overlap between 5–40 m.o. degus. **(L)** Inter-group S1 layer analysis shows juvenile degus have significantly lower PNN levels than their older counterparts in all S1 layer except L1, which exhibited minimal-to-no PNNs. Error bars represent SEM; Kruskal–Wallis test followed by *post-hoc* Dunn's test: ^trend^*p* < 0.1, *p < 0.05, **p < 0.01, ***p < 0.001, ****p < 0.0001.

The fourth and final cortical area examined was the primary somatosensory cortex (S1; Figure 5), chosen as a representative region of primary sensory cortex to balance “higher-level” areas of frontal, temporal, and parietal cortices. Juvenile degus exhibited low-PNN but high levels of microglia, which gradually inverted in older animals (layer 4, Figure 5A). Juvenile degus had significantly lower PNN levels than adolescent and older degu age groups [*H*_(3, 28)_ = 19.55, *p* = 0.0002, KW ANOVA; *p* = 0.009, *p* = 0.0001, Dunn's test; Figure 5E] and significantly higher Iba intensity levels than all older age groups [*H*_(3, 28)_ = 17.58, *p* = 0.0005, KW ANOVA; *p* = 0.02, *p* = 0.005, *p* = 0.0012, Dunn's test; Figure 5H]. This pattern yielded a positive linear relationship for PNNs (*R*^2^ = 0.28, *p* = 0.001) and a negative relationship for Iba1 relative intensity with age (*R*^2^ = 0.30, *p* = 0.0009; Figures 5F, I). PNNs had a younger sigmoid inflection age (3.4 months) than microglia (4.9 months), although the difference in these inflection ages was smaller than in other cortical regions (Figures 5G, J). When both plasticity markers are considered together, juvenile degus cluster relatively distinctly from a larger group of adolescent and adult animals (Figure 5K).

Closer analysis of degu S1 revealed PNNs were expressed differentially between cortical layers. Deep layer 6 had the greatest PNN density, which gradually decreased in more superficial layers (L6 > L5 > L4 > L2/3 > L1; Figures 5B–D). This pattern of S1 laminar PNN expression differs from what is seen in humans, mice, rats, and Mongolian gerbils (Brückner et al., 1994; Hausen et al., 1996; Köppe et al., 1997; Ueno et al., 2019; Venturino et al., 2021; de Medeiros Brito et al., 2022). Detailed analysis of one of the juvenile degus beginning to express PNNs showed they do so predominantly in layer 5/6, suggesting PNN establishment and maturation in the degu starts in the deeper layers and expands upwards (Figures 5B1, C1, D1). Intergroup layer analysis revealed juvenile degus had reduced PNNs in all layers except layer 1 (which had very low PNN densities across all degu ages) when compared to older counterparts (Figure 5L).

### 3.3 Perineuronal nets and microglia expression patterns in the degu hippocampus

**Figure 6:**
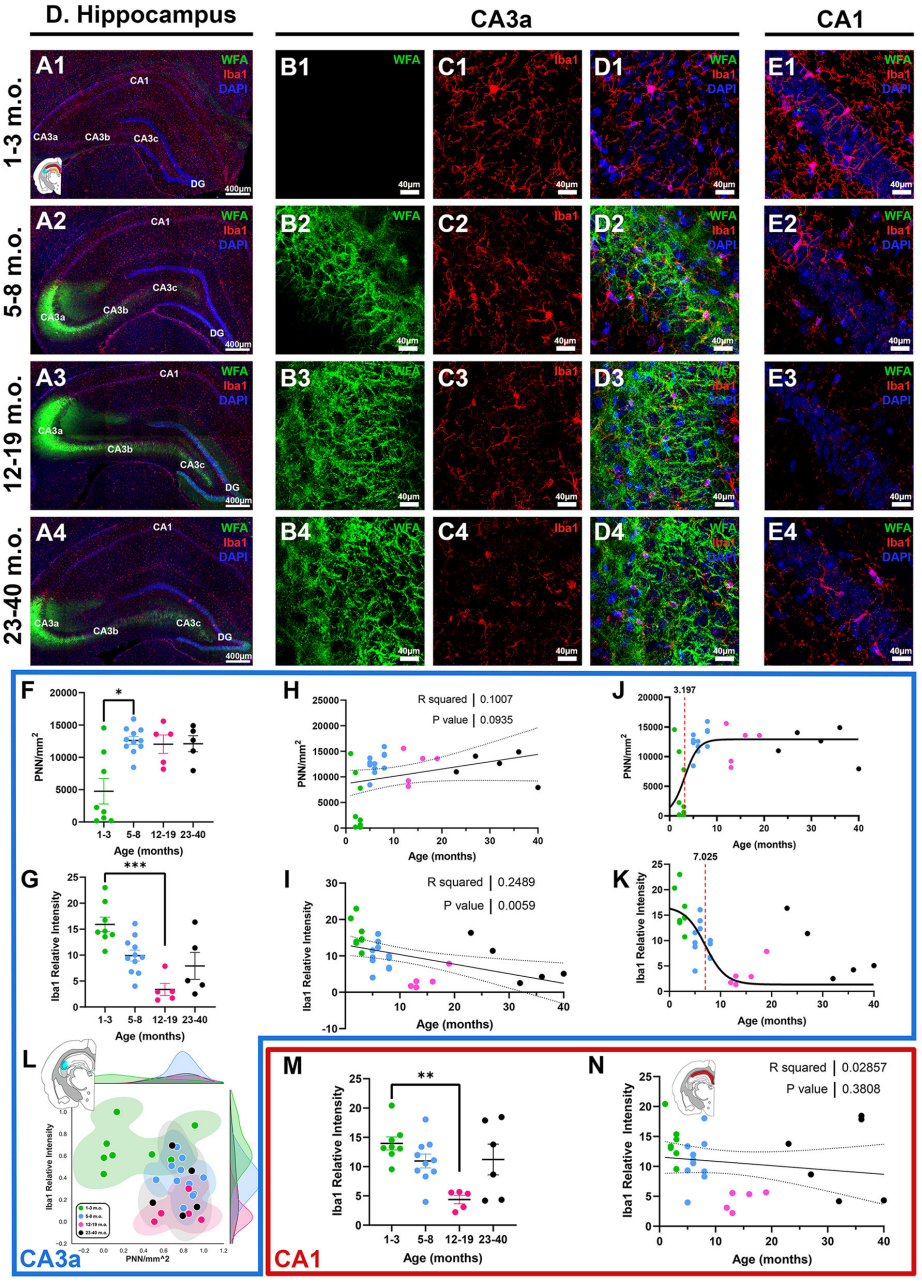
Degus possess a distinct hippocampal perineuronal net (PNN) expression pattern characterized by intense CA3a signal and minimal-to-no signal in CA1. **(A–E)** Immunofluorescence confocal micrographs showing PNNs (WFA, green) and microglia (Iba1, red) in the entire dorsal hippocampus **(A)**, CA3a **(B, C)**, and CA1 **(E)** of 1–3 m.o. (juvenile, 1st row), 5–8 m.o. (adolescent, 2nd row), 12–19 m.o. (younger adult, 3rd row), and 23–40 m.o. (older adult, 4th row) degus. **(F)** PNN density quantification showed a significant difference between 1–3 m.o. degus and 5–8 m.o. degus (*p* = 0.029). **(G)** 1–3 m.o. degus exhibit increased CA3a microglia Iba1 relative intensity than 12–19 m.o. counterparts (*p* = 0.0005). **(H, I)** Linear regression analysis found a statistically trending positive correlation between PNN/mm^2^ and age [**(H)**, *R*^2^ = 0.1; *p* = 0.093], while Iba1 relative intensity had a significant negative correlation with age [**(I)**, *R*^2^ = 0.24; *p* = 0.0059]. **(J, K)** Sigmoid curve analysis revealed PNNs have an earlier inflection age (3.19 months) than microglia (7.02 months) in degu CA3a. **(L)** Normalized PNN/mm^2^ and Iba1 relative intensity 2D plot revealed some 1–3 m.o. degus express increased PNN levels in CA3a and clustered closer to post-puberty age groups. **(M)** Age group analysis showed 12–19 m.o. degu have significantly lower CA1 Iba1 relative intensity levels than 1–3 m.o. degus. **(N)** Linear regression analysis found no significant correlation between CA1 microglia Iba1 intensity and age. Error bars represent SEM; Kruskal–Wallis test followed by *post-hoc* Dunn's test: ^trend^*p* < 0.1, *p < 0.05, **p < 0.01, ***p < 0.001.

The hippocampus has been historically a target for studies of lifespan plasticity and learning and memory (McGaugh, 2000; Stickgold, 2005; Andersen et al., 2006). Different subdivisions of the hippocampus serve different functions and processing steps, including those of the classical trisynaptic loop (DG, CA3, and CA1), the subiculum (SUB), as well as further subdivisions along the proximo-distal axis, including CA3c (closest to DG), CA3b, and CA3a (closest to CA1) (Lorente de Nó, 1934; Sun et al., 2017; Lin et al., 2021), offering an opportunity to examine more detailed circuitry associated with PNN and microglia. CA3a exhibited the strongest PNN signal (Figures 6A, B), characterized by diffuse PNN structures outlining cell soma and neuropil, which contrasted from the more structured cortical PNNs that enwrap larger portions of the proximal dendrites. Diffuse PNNs were also observed in DG, while PNNs in CA3b and CA3c enwrapped greater portions of the proximal dendrites. Juvenile degus exhibited significantly lower CA3a PNN signals compared to adolescents [*H*_(3, 25)_ = 9.208, *p* = 0.026, KW ANOVA; *p* = 0.029, Dunn's test; Figure 6F], and there was a statistical trend toward a significant positive linear relationship between PNN density and age (*R*^2^ = 0.1, *p* = 0.09; Figure 6H). The CA3a 2D plot showed some juveniles pooling closer to post-sexual maturity age degus along the PNN axis, possibly indicating early CA3a PNN development (Figure 6L). Similar to what was seen in cortical regions RSC and S1, microglia Iba1 intensity had a significant negative linear regression (*R*^2^ = 0.24, *p* = 0.005; Figure 6I), with juvenile degus exhibiting robust Iba1 relative intensity that was significantly higher than that of younger adults [*H*_(3, 25)_ = 16.22, *p* = 0.001, KW ANOVA; *p* = 0.0005, Dunn's test; Figures 6C, G]. Sigmoid curves in CA3a showed a similar pattern as in cortical regions, with PNNs having an earlier inflection age (3.1 months) than microglia (7.0 months; Figures 6J, K). Although showing minimal-to-no PNNs, juvenile degu CA1 expressed strong Iba1 relative intensities similar to those seen in other brain regions, which were significantly higher than in younger adults [*H*_(3, 25)_ = 10.75, *p* = 0.013, KW ANOVA; *p* = 0.006, Dunn's test; Figures 6E, M, N].

**Figure 7:**
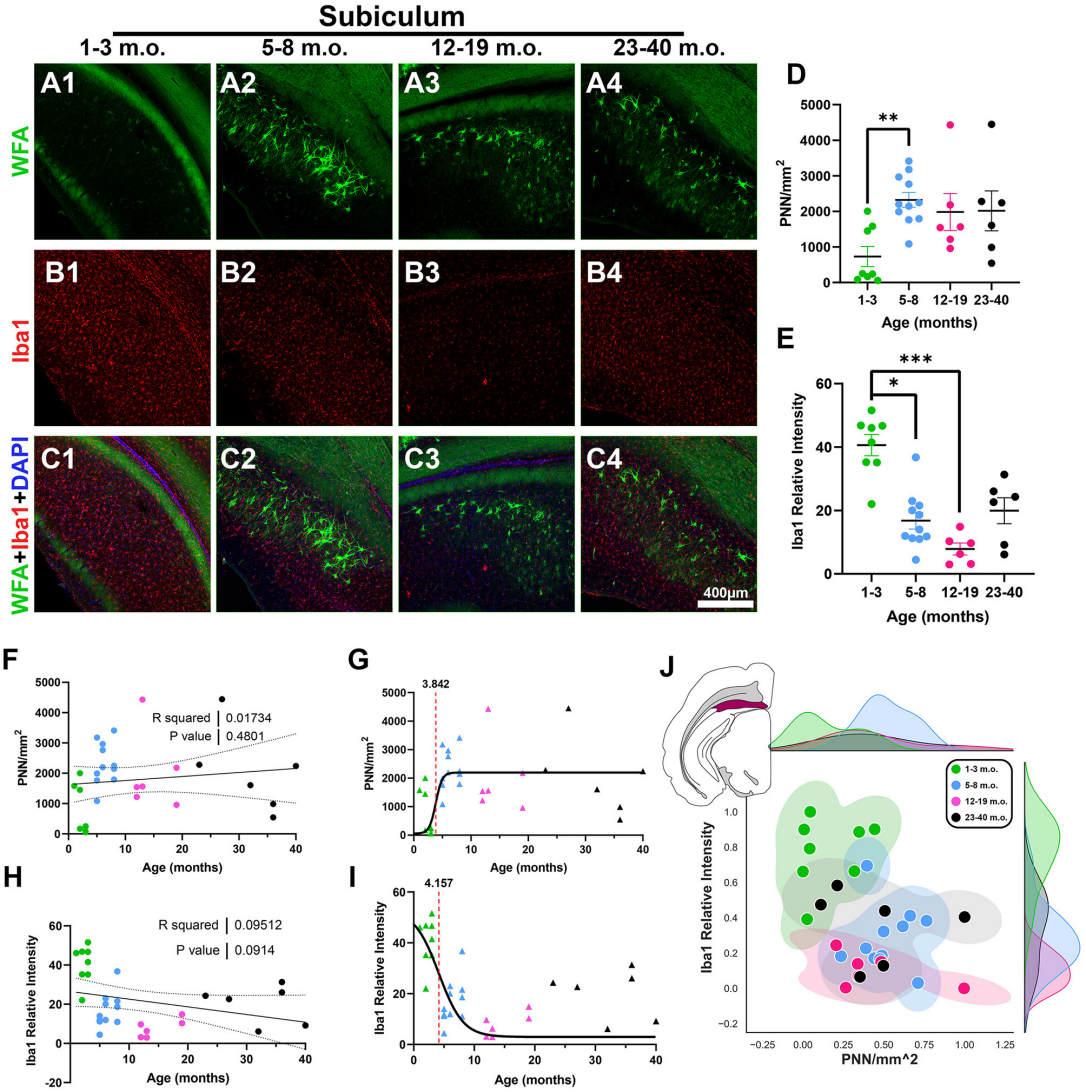
Degu subiculum (SUB) exhibits decreasing levels of microglia with age and similar microglia and perineuronal net (PNN) plasticity timecourse inflection ages. **(A–C)** Confocal micrographs showing PNNs (WFA, green) and microglia (Iba1, red) in the SUB of 1–3 m.o. (juvenile) **(A1, B1, C1)**, 5–8 m.o. (adolescent) **(A2, B2, C2)**, 12–19 m.o. (younger adult) **(A3, B3, C3)**, and 23–40 m.o. (older adult) **(A4, B4, C4)** degus. **(D)** PNNs per mm^2^ quantification identified a significant difference between 1–3 m.o. degus and 5–8 m.o. degus (*p* = 0.0052). **(E)** 1–3 m.o. degus showed increased SUB microglia signal when compared to 5–8 m.o./12–19 m.o. degus (*p* = 0.016, *p* = 0.0002, and *p* = 0.02 respectively). **(F, H)** Linear regression analysis found no significant correlation between PNN/mm^2^ and age [**(F)**, *R*^2^ = 0.02; *p* = 0.439] while Iba1 relative intensity exhibited a statistical trend toward a negative correlation [**(H)**, *R*^2^ = 0.095; *p* = 0.091]. **(G, I)** Sigmoid curve analysis revealed SUB exhibits similar PNN (3.84) and microglia (4.15) inflection ages, unlike what is seen in most of the other analyzed brain regions. **(J)** Normalized individual data points for 1–40 m.o. degus in PNN/mm^2^ and Iba1 relative intensity space showed some intermixing between all age groups. Error bars represent SEM; Kruskal–Wallis test followed by *post-hoc* Dunn's test: ^trend^*p* < 0.1, *p < 0.05, **p < 0.01, ***p < 0.001.

Juvenile degus exhibited significantly lower SUB PNN densities compared to adolescent degus [*H*_(3, 27)_ = 11.13, *p* = 0.0095, KW ANOVA; *p* = 0.0052, Dunn's test; Figures 7A, D], although no significant PNN linear trend was found with age progression (*R*^2^ = 0.017, *p* = 0.48; Figure 7F). This lack of age-related PNN changes was reflected in the fraction of juvenile degus mixed with post-sexual maturity degus in the 2D plot (Figure 7J). Robust Iba1 signal was seen in juveniles that was significantly higher than adolescent and younger adult age groups [*H*_(3, 27)_ = 18.32, *p* = 0.0004, KW ANOVA; *p* = 0.016, *p* = 0.0002, Dunn's test; Figures 7B, E] with a statistically trending negative linear relation with age (*R*^2^ = 0.09, *p* = 0.09; Figure 7H). Sigmoid analysis showed PNN and microglia have, in contrast to previous regions, similar inflection ages (3.8 and 4.1 months, respectively; Figures 7G, I). The SUB 2D plot showed some level of intermixing between all degu age groups, although juveniles mostly populated the upper-left portion of the plot (Figure 7J).

### 3.4 Perineuronal nets and microglia in the degu basolateral amygdala

**Figure 8:**
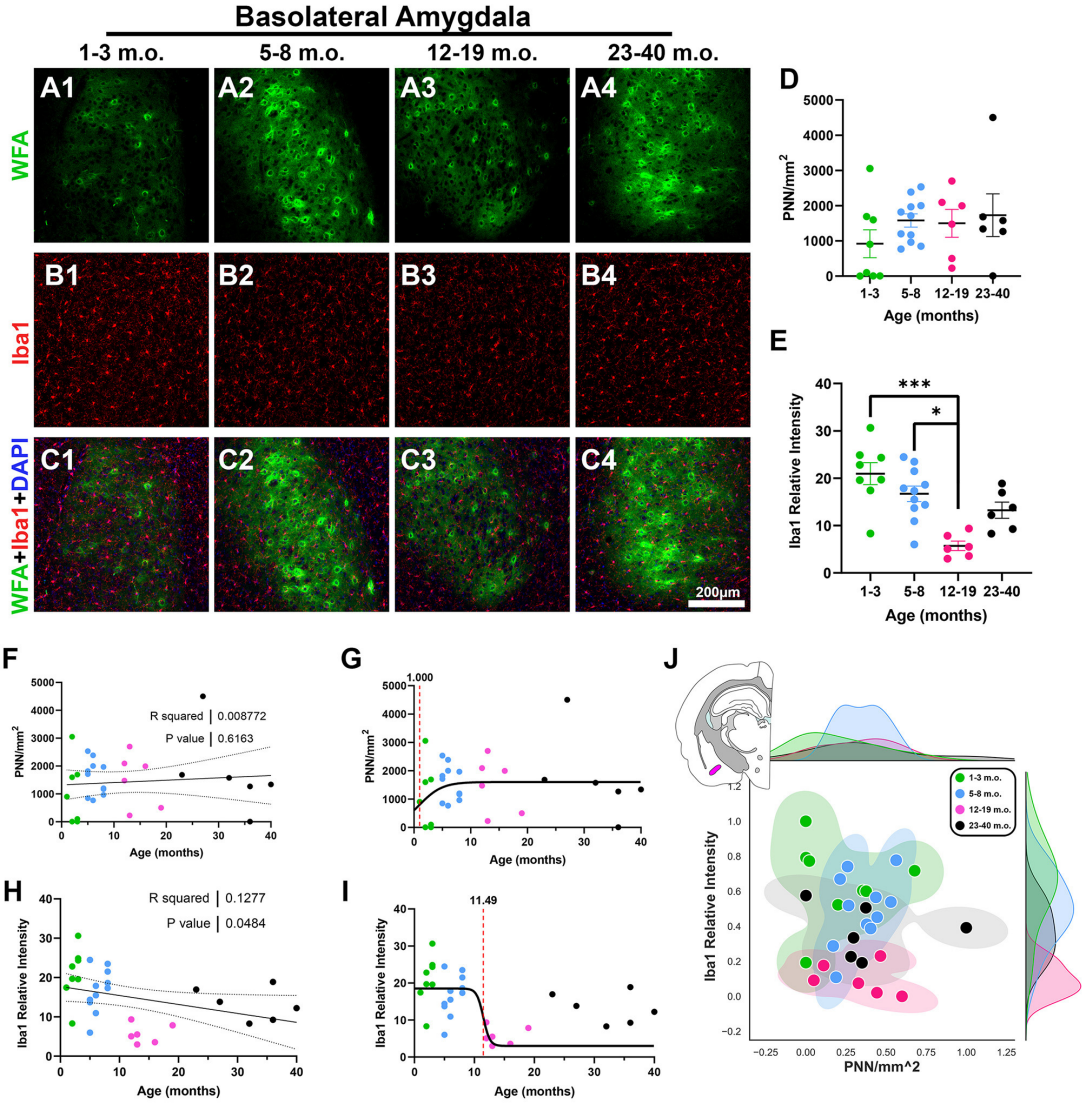
Degu basolateral amygdala (BLA) shows no age-related perineuronal net (PNNs) changes, while microglia decrease with age progression. **(A–C)** Immunofluorescence confocal micrographs of PNNs (WFA, green) and microglia (Iba1, red) in the BLA of 1–3 m.o. (juvenile) **(A1, B1, C1)**, 5–8 m.o. (adolescent) **(A2, B2, C2)**, 12–19 m.o. (younger adult) **(A3, B3, C3)**, and 23–40 m.o. (older adult) **(A4, B4, C4)** degus. **(D)** Quantification of PNN densities found no significant differences between age groups. **(E)** 12–19 m.o. degus showed significantly decreased microglia signal measured by Iba1 relative intensity than younger 1–3 m.o. and 5–8 m.o. age groups (*p* = 0.0005 and *p* = 0.016, respectively). **(F, H)** Linear regression analysis found no significant correlation between PNN/mm^2^ **(F)** and age progression (*R*^2^ = 0.0087; *p* = 0.61), while Iba relative intensity **(H)** exhibited a significant negative correlation (*R*^2^ = 0.1277; *p* = 0.048). **(G, I)** PNN sigmoid inflection age (1 month) occurred earlier than microglia (11.49 months) in the BLA. **(J)** Normalized PNN/mm^2^ and Iba1 relative intensity 2D plot from 1–40 m.o. degus shows extensive intermixing between all age groups. Error bars represent SEM; Kruskal–Wallis test followed by *post-hoc* Dunn's test: ^trend^*p* < 0.1, *p < 0.05, ***p < 0.001.

We next examined the developmental profile of the basolateral amygdala (BLA), in part because of its known role in social-emotional information processing (Phelps and LeDoux, 2005), combined with the importance of social behavior to degu behavioral ecology (Ebensperger et al., 2004). We found differences in BLA microglia, where younger adults had a significantly lower Iba1 intensity than juvenile and adolescent degus [*H*_(3, 27)_ = 16.59, *p* = 0.0009, KW ANOVA; *p* = 0.0005, *p* = 0.016, Dunn's test; Figures 8B, E], and an overall significantly decreasing linear regression (*R*^2^ = 0.12, *p* = 0.048; Figure 8H). However, no significant changes in BLA PNN densities were observed (Figures 8A, D, F), which is illustrated in the BLA 2D plot where all 4 groups had some level of mixing (Figure 8J). The inflection age was much smaller in PNN (1 month) than in microglia (11.4 months; Figures 8G, I), suggesting differing PNN and microglia neuroplasticity timecourses in the BLA.

### 3.5 Perineuronal nets and microglia in the degu subcortical thalamic reticular nucleus

**Figure 9:**
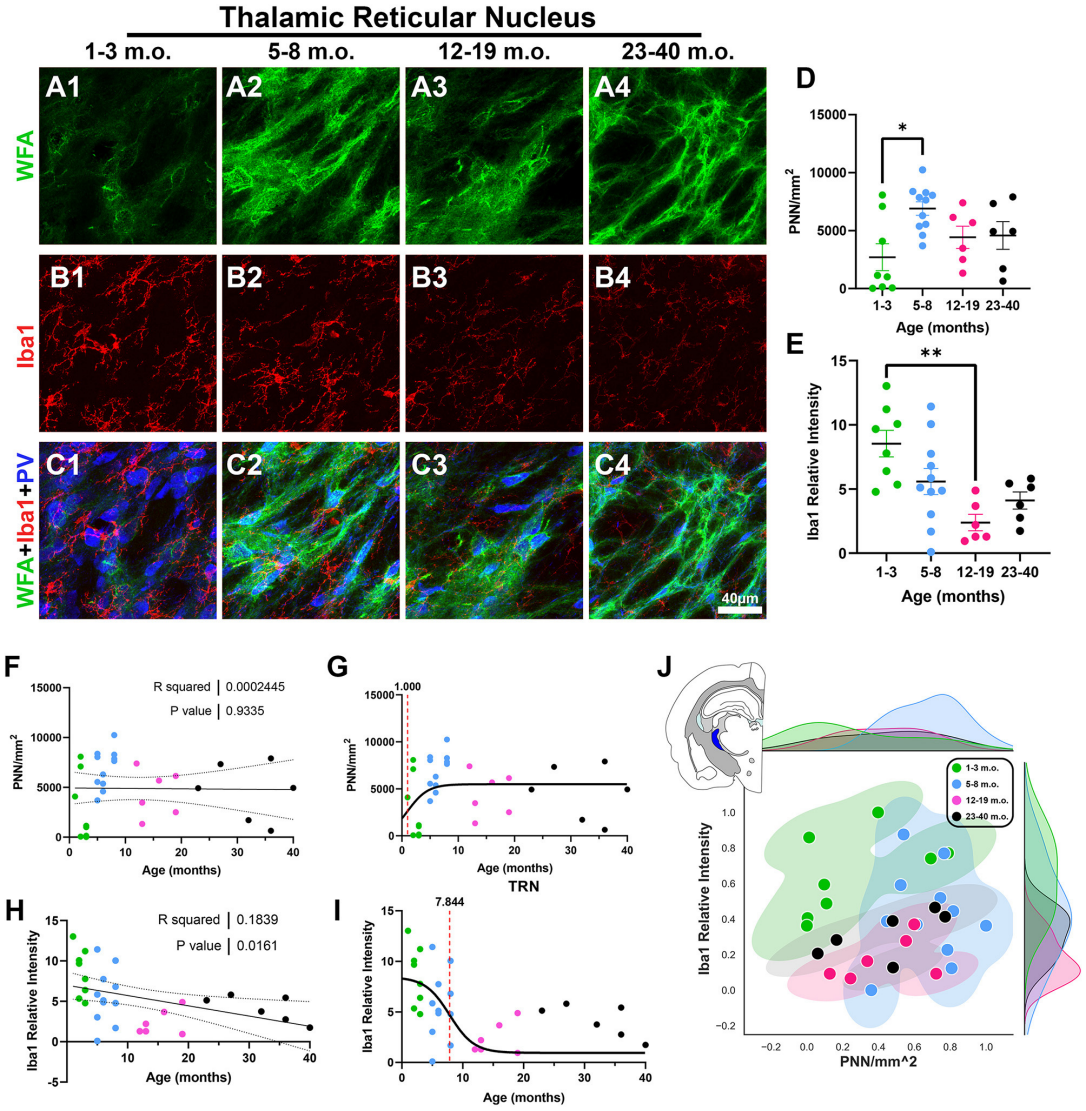
Degu thalamic reticular nucleus (TRN) exhibits decreasing microglia levels with age and differing perineuronal net (PNN) densities between juvenile and adolescent life phases. **(A–C)** Confocal micrographs showing PNNs (WFA, green), microglia (Iba1, red), and parvalbumin+ interneurons (PV, blue) in the TRN of 1–3 m.o. (juvenile) **(A1, B1, C1)**, 5–8 m.o. (adolescent) **(A2, B2, C2)**, 12–19 m.o. (younger adult) **(A3, B3, C3)**, and 23–40 m.o. (older adult) **(A4, B4, C4)** degus. **(D)** PNN quantification found a significant difference between 1–3 m.o. and 5–8 m.o. degus (*p* = 0.003). **(E)** 1–3 m.o. degu TRN showed increased microglia levels than 12–19 m.o. degu TRN (*p* = 0.02). **(F, H)** Linear regression analysis of PNN/mm^2^ **(F)** and Iba1 relative intensity **(H)** with degu age. PNN/mm^2^ showed no significant correlation with age (*R*^2^ = 0.0002; *p* = 0.933) while Iba1 relative intensity exhibited a significant negative correlation with age (*R*^2^ = 0.183; *p* = 0.016). **(G, I)** Sigmoid data curve fittings identified an earlier PNN inflection age (1 month) than microglia (7.84 months) in the degu's TRN. **(J)** Normalized PNN/mm^2^ and Iba1 relative intensity 2D space displayed extensive intermixing between post-puberty age groups and some mixing between 1–3 m.o. and 5–8 m.o. degus. Error bars represent SEM; Kruskal–Wallis test followed by *post-hoc* Dunn's test: ^trend^*p* < 0.1, *p < 0.05, **p < 0.01.

As the thalamus exhibits mechanisms of plasticity that differ from those seen in cortical regions (Kaas, 1999), we next looked at the thalamic reticular nucleus (TRN). The TRN possesses strong PNN signal and has roles in sensory processing, arousal, and cognition (Li et al., 2020). Further, the TRN is composed of GABAergic neurons that highly express the calcium-binding protein parvalbumin (PV), which are the type of neurons most likely to be enwrapped by PNNs (Zikopoulos and Barbas, 2006; Liu et al., 2017; Fawcett et al., 2019). In addition to WFA and Iba1, we immunostained against PV to clearly identify the degu TRN. We found that adolescent degus express robust PNN densities, which were significantly higher than in the juvenile degu [*H*_(3, 27)_ = 9.017, *p* = 0.029, KW ANOVA; *p* = 0.0209, Dunn's test; Figures 9A, D]. However, linear regression did not show any significant PNN changes with age (*R*^2^ = 0.0002, *p* = 0.93; Figure 9F). TRN microglia showed a significant negative linear regression (*R*^2^ = 0.18, *p* = 0.016; Figure 9H), with juvenile degus exhibiting a significantly higher Iba1 relative intensities than younger adult degus [*H*_(3, 27)_ = 12.61, *p* = 0.0056, KW ANOVA; *p* = 0.0031, Dunn's test; Figures 9B, E]. The TRN sigmoid inflection age was smaller in PNN (1 month) than microglia (7.8 months; Figures 9G, I), similar to what was seen in most of the examined brain regions. The 2D plot shows some mixing between all groups, with adolescent degus showing the broadest territorial coverage (Figure 9H).

### 3.6 Adolescent degus exhibit adult-like PNNs, but intermediate levels of microglia

**Figure 10:**
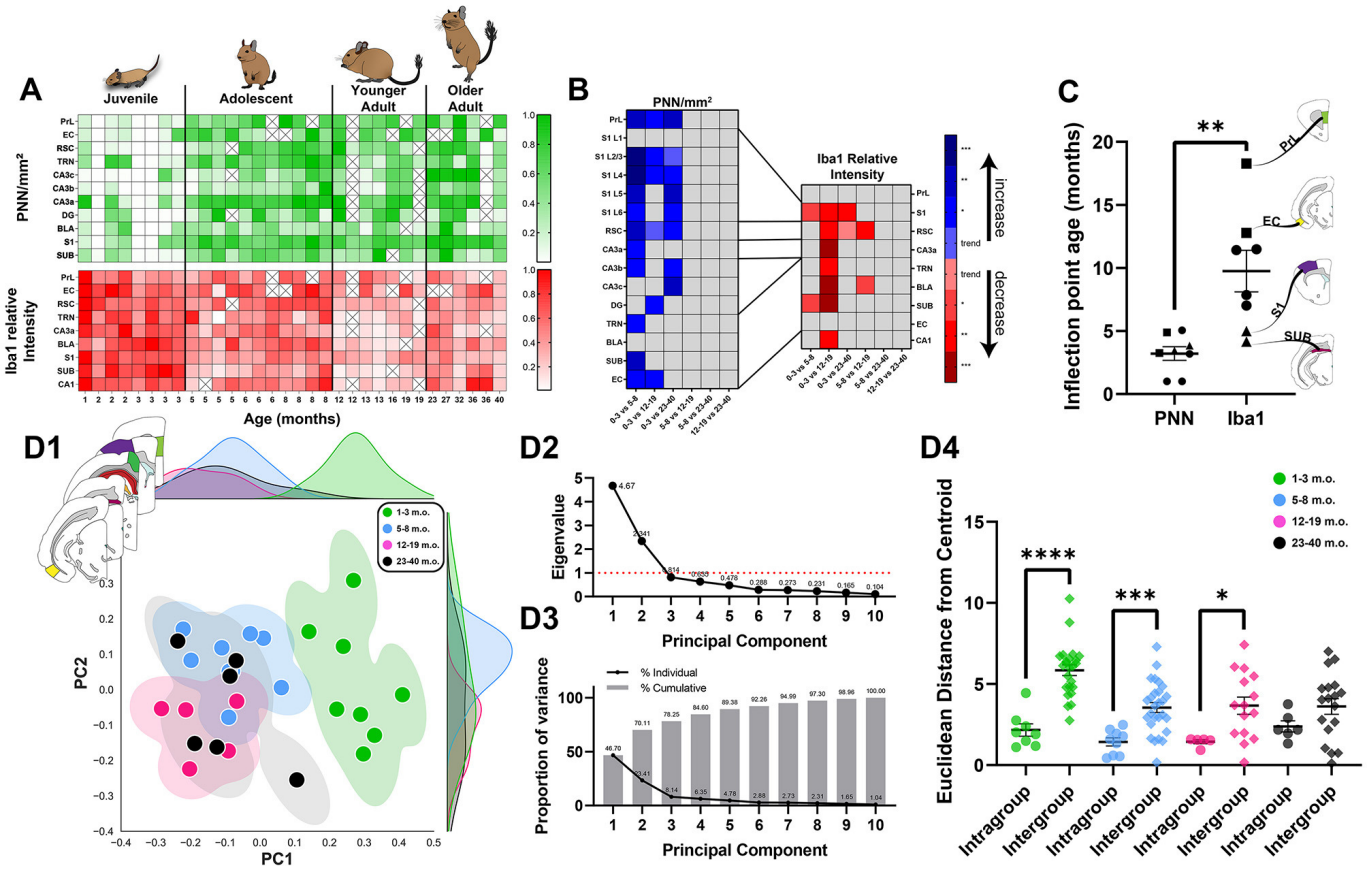
The maturing degu forebrain exhibits differing neuroplasticity features in juvenile, adolescent, and adult life stages. **(A)** Normalized perineuronal net (PNN) densities and microglia Iba1 relative intensities in 1–40 m.o. degus across the investigated brain regions. **(B)** Summary matrix with statistically significant PNN/mm^2^ and Iba1 relative intensity age group differences across brain regions. All significant PNN differences involved comparisons with juvenile (1–3 m.o.) degus, while microglia showed some differences between adolescent (5–8 m.o.) and younger adult (12–19 m.o.) groups. Box colors indicate direction of change with increasing age (blue—increase; red—decrease), and intensity of color denotes *p*-value range (Kruskal–Wallis test followed by *post-hoc* Dunn's test). **(C)** Summary of sigmoid curve inflection ages for PNN densities and microglia Iba1 intensities. PNN inflection ages were significantly younger than microglia inflection ages across the studied brain regions (*p* = 0.0011, Mann–Whitney test). Early microglia transition regions, SUB and S1, are denoted with triangles, while late microglia transition regions, PrL and EC, are denoted with squares. **(D)** Principal component analysis (PCA) of 1–40 m.o. degu PNN densities and microglia Iba1 relative intensities reveal juvenile degus (1–3 m.o) cluster separately from post-puberty (5–40 m.o.) degus. Adolescents and younger adults showed small territorial overlap between them, while older adult (23–40 m.o.) degus spanned a larger region overlapping with adolescent and younger adult degus **(D1)**. The top two principal components used to plot PCA data, with eigenvalues of 4.67 and 2.34 **(D2)**, accounted for >70% of the dataset variance **(D3)**. Euclidean distance analysis **(D4)** on PCA data shows juvenile, adolescent, and younger adult age group clusters have significantly smaller (*p* = 0.0001, *p* = 0.0003, *p* = 0.01, Mann–Whitney test) distances from their assigned (intragroup) age group's centroid than from that of other age groups (intergroup), highlighting the distinct juvenile to younger adult age clusters identified by PCA. Older degus showed no significant difference between intra- and intergroup Euclidean distances (*p* = 0.136, Mann–Whitney test). Error bars represent SEM; ^trend^*p* < 0.1, *p < 0.05, **p < 0.01, ***p < 0.001, ****p < 0.0001.

The principle goal of the present study was to assess changes in plasticity markers over brain maturation. We found a general pattern of high microglia but low PNN in early age that gradually inverted with brain maturation, with the majority of statistically significant differences involving comparisons with the juvenile age group (Figures 10A, B). However, both qualitative observation across the brain (Figure 1) and detailed analysis of specific regions (Figures 2–9) suggested that the two markers chosen, PNN and microglia, do not follow the same developmental timecourse. To quantify the transitions in PNN and microglia levels from juvenile to adulthood, logistic sigmoid functions were fitted to age datasets and analyzed for curve inflection points in all brain regions.

Data were initially fitted using minimal constraints to ensure sigmoid plot shapes with inflection points/ages occurring in the analyzed degu ages (1–40 months). Results showed PNN inflection points (median = 3.3 months) were significantly lower (*p* = 0.0003, Mann–Whitney test) than those seen in Iba1 relative intensities (median = 12.6 months). However, variance in older age groups resulted in some brain regions exhibiting poor curve fittings, which motivated us to further constrain the model to ensure a more accurate fitting capable of identifying the youth-to-adult transition in the data (panels G, I in Figures 2–4, 7–9; panels G, J in Figure 5; panels J, K in Figure 6). Using these settings, we continued to observe a statistically significant difference (*p* = 0.0011, Mann–Whitney test), with PNNs (median = 3.3 months) showing younger inflection point ages than microglia (median = 9.6 months; Figure 10C, latest microglia transition brain regions in squares, earliest in triangles). Two regions known to process abstract and context information, PrL and EC, showed the latest microglia transitions (in squares) and the primary sensory area, S1, was among the earliest to transition (in triangles). Microglia inflection ages also had a significantly higher variance (σ^2^ = 21.7) than PNN inflection ages (σ^2^ = 2.3; ^**^p = 0.0087, *F*-test).

Principal component analysis (PCA) was conducted to further analyze the different age degu populations. Juvenile degus isolated themselves on the right side of the PCA plot, while post-puberty age degus intermix on the left (Figure 10D1). Juvenile, adolescent, and younger adult degus seem to occupy separate territories in the multi-region, PCA space, while older adult degus are more dispersed. PCA data was plotted using the 2 eigenvectors with largest eigenvalues, which accounted for >70% of the variance (Figures 10D2, D3). Euclidean distances from age group centroids were calculated for all degu data points. All age groups, except older age degus (*p* = 0.13, Mann–Whitney test), had significantly smaller Euclidean distances from their assigned age group than from others (juvenile: *p* = 0.0001, adolescent: *p* = 0.0003, younger adult: *p* = 0.01, Mann–Whitney test), highlighting the distinction between clusters of juvenile, adolescent, and younger (but not older) adult age groups (Figure 10D4). MANOVA analysis found a significant association between degu age groups and their corresponding PCA datapoints [*V* = 1.0536, *F*_(6, 48)_ = 8.91, *p* = 1.54 × 10^−6^, Pillai's trace]. *Post-hoc* univariate ANOVA analysis was significant for principal component 1 [PC1, which had positive loadings for brain regions' PNN densities and negative loading values for Iba1 intensities; *F*_(3, 24)_ = 32.709; *p* = 1.209 × 10^−8^, ANOVA], and trended toward significance in PC2 [which had negative loadings for both PNN and Iba1 levels; *F*_(3, 24)_ = 2.668; *p* = 0.0704, ANOVA]. Subsequent Tukey HSD tests found significant differences between juvenile and all older age groups' PC1 values (*p* = 0.001 for all 3, Table 3). Adolescent degus had a statistically trending difference in PC2 values when compared to younger adult degus (*p* = 0.0701, Table 3).

**Table 3:** MANOVA *post-hoc* Tukey HSD (honestly significant difference) statistical analysis of principal component analysis data.

***Post-hoc*** **Tukey's HSD multiple comparisons**				
**Dataset**	**Age group 1**	**Age group 2**	**Mean** Δ	***p*** **-value**
Principal component 1	Juvenile (0–3)	Adolescent (5–8)	−0.357	0.001
Principal component 1	Juvenile (0–3)	Y. adult (12–19)	−0.446	0.001
Principal component 1	Juvenile (0–3)	O. adult (23–40)	−0.374	0.001
Principal component 1	Adolescent (5–8)	Y. adult (12–19)	−0.088	0.350
Principal component 1	Adolescent (5–8)	O. adult (23–40)	−0.017	0.900
Principal component 1	Y. Adult (12–19)	O. adult (23–40)	0.071	0.587
Principal component 2	Juvenile (0–3)	Adolescent (5–8)	0.060	0.747
Principal component 2	Juvenile (0–3)	Y. adult (12–19)	−0.126	0.337
Principal component 2	Juvenile (0–3)	O. adult (23–40)	−0.070	0.725
Principal component 2	Adolescent (5–8)	Y. adult (12–19)	0.186	0.070
Principal component 2	Adolescent (5–8)	O. adult (23–40)	0.1306	0.245
Principal component 2	Y. adult (12–19)	O. adult (23–40)	0.056	0.884

Following MANOVA statistical analysis of principal component analysis (PCA) data from perineuronal net densities and Iba1 relative intensities, *post-hoc* Tukey HSD tests of all degu age groups head-to-head permutations were conducted to identify differences between age groups. Mean differences in regards to specific principal components (1 or 2, which together accounted for >70% of PCA variance) are detailed for each comparison along with their corresponding *p*-value.

## 4 Discussion

The current study provides an extensive characterization of PNNs (regional/laminar details in Table 4) and microglia in the developing postnatal degu brain. We find degu PNN and microglia expression patterns follow typical mammalian developmental plasticity, where juvenile, sexually immature, subjects exhibit low PNN levels coupled with a large microglia presence that inverts with age progression and circuit maturation (Yamada and Jinno, 2013; Brust et al., 2015; Lenz and Nelson, 2018; Rogers et al., 2018; Menassa et al., 2022) (Figure 10A). Our analysis of four degu age groups shows, perhaps unsurpsingly, that PNN and microglia particularly distinguish the juvenile life phase (Figure 10B), consistent with these two plasticity mediators playing unique roles in development.

**Table 4:** Perineuronal net localization and density data summary across degu age groups and brain regions.

**Perineuronal nets/mm** ^**2**^						
**Brain region**	**PNN localization**	**Measure**	**Juvenile (1–3 m.o.)**	**Adolescent (5–8 m.o.)**	**Younger adult (12–19 m.o)**	**Older adult (23–40 m.o.)**
PrL	L5	Mean	275	1,826	1,787	1,906
		Standard deviation	240.7	581.1	295.7	854.6
EC	L2–4	Mean	1,062	3,944	4,645	3,504
		Standard deviation	15,18	1,791	875.3	2,980
RSC	L2, L2/3, L5	Mean	429	3,608	3,203	3,659
		Standard deviation	723.2	1,505	1,897	2,039
TRN	Entire structure	Mean	2,704	6,902	4,422	4,574
		Standard deviation	3,297	1,954	2,347	2,921
SUB	Pyramidal layer	Mean	729.6	2,323	1,982	2,017
		Standard deviation	803.3	693.5	1,268	1,373
BLA	Entire structure	Mean	920	1,580	1,499	1,730
		Standard deviation	1,122	620.3	965.2	1,486
S1	L6 > L5 > L4 > L2/3 > L1	Mean	1,008	2,864	2,736	3,518
		Standard deviation	942.4	674.2	553.3	519.3
CA3a	Pyramidal cell layer	Mean	4,750	12,607	12,034	12,100
		Standard deviation	5,570	2,001	3,170	2,763
CA3b	Pyramidal cell layer	Mean	399.4	2,474	2,312	2,926
		Standard deviation	533	1,910	1,775	1,728
CA3c	Pyramidal cell layer	Mean	441.9	1,496	1,606	3,311
		Standard deviation	854.7	919.1	866.4	1,190
DG	Granule cell layer	Mean	1,379	4,482	6,177	3,649
		Standard deviation	2,153	2,967	3,227	3,354
CA1	Minimal-to-no signal	Mean	—	—	—	—
		Standard deviation	—	—	—	—

Summary of perineuronal net localization (in adolescents and adults), density means, and standard deviations across brain regions in the 4 studied degu age groups (juveniles, adolescents, younger adults, and older adults).

Although PNN and microglial markers were largely anti-correlated, the ways in which they were not revealed unique properties in adolescence. Assuming a linear relationship between age and PNN density, we expected to see at least a few statistical differences between adolescent and adult PNN levels. This was not the case, as adolescent PNN densities were similar to those of adult age groups (Table 4). Sigmoid curve analysis articulated the differences between the plasticity markers, as PNNs had significantly younger inflection ages (median of 3.3 months) than microglia (median of 9.6 months), indicating PNNs are reaching adult-like states quicker than microglia (Figure 10C). PCA analysis suggested the presence of juvenile, adolescent, and younger adult degu clusters (Figure 10D). Subsequent PCA data analysis found juvenile degus were distinct from older age groups along PC1, as might be expected, but along PC2 there was a statistically trending difference between adolescents and younger adults (Table 3). These results hint at a unique state of neuroplasticity in adolescence where nonlinear, possibly switch-like, ECM reorganization starkly stabilizes synapse architecture, while highly expressing Iba1 microglia are possibly still pruning/remodeling synapses at levels higher than in adulthood (Carulli 2021). These transitional differences suggest microglia may offer a window into developmental plasticity timecourse differences across the brain. As previous studies found a large number of mental health conditions manifest in childhood or adolescence (Kessler et al., 2007; Solmi et al., 2022), with many of them exhibiting dysfunctional plasticity (Tatti et al., 2017; Sellgren et al., 2019), future research looking at neuroplasticity abnormalities during pre- and early pubescence could provide valuable insights into our understanding and treatment of these conditions.

Although most studied brain regions showed increased PNNs after sexual maturity, microglia changes with age were highly variable across regions. This likely reflects region-specific developmental plasticity timecourses necessary for region-specific functional purposes. The PrL is one such area. Although Iba1 intensities showed no significant differences between age groups, the combination of a statistically significant negative regression and a very late inflection age suggests PrL microglial changes took place relatively gradually, and later than in other regions. This suggests the rodent PrL, a subdivision of the rostral cingulate cortex, is late to develop—not unlike the rostral prefrontal cortex in humans (Dumontheil et al., 2008). The PrL is thought to use high-level, abstract information to support task-relevant behavioral schemas and their updating (Delatour and Gisquet-Verrier, 2000; Rich and Shapiro, 2007; Tse et al., 2011; Euston et al., 2012; Broschard et al., 2021). It seems likely these functions depend on the development of other percepts and action representations; in other words, network refinement may presuppose network stabilization in other sensory and motor regions. The same may be true of the EC, an area that performs complex operations on highly integrated perceptual and cognitive information (Garcia and Buffalo, 2020). S1, in contrast, receives direct inputs from the sensory thalamus, and likely forms representations of percepts during early stages of development. One region that does not succinctly fit into this framework is the SUB, which exhibited a relatively earlier microglia inflection point, like S1, but is often considered alongside EC due to the type of information it processes (O'Mara et al., 2009). Further studies will be useful in understanding why networks may stabilize early in the SUB.

We found that the BLA was the only region to break the pattern of reduced juvenile PNNs that gave-way to higher PNN levels in adolescence. It is unclear if this was due to early PNN development in the BLA, absence of PNN development in a subset of adults, or a more general trend for less reliable PNN formation in this region. While the amygdala is thought to be fully developed at birth, it continues to undergo functional changes during adulthood (Avino et al., 2018). Half of the juvenile BLA's assayed in this study showed adult-like PNN levels, consistent with the possibility of early PNN formation in the BLA (Figure 8). Given the precocial nature of degus, it may make sense for some networks, like those of a central emotional hub, to stabilize early, enabling early adaptive responses to emotional stimuli and offering a foundation for other brain networks to follow.

Our analysis shows degu S1 has a distinct laminar pattern of PNN expression, with minimal-to-no PNN density in layer 1 that gradually increases to its greatest density in layer 6 (Figure 5). This differs from other species, such as the mouse, rat, and Mongolian gerbil, where PNN is highest in layer 4 (Brückner et al., 1994; Köppe et al., 1997; Ueno et al., 2019; Venturino et al., 2021; de Medeiros Brito et al., 2022; Mascio et al., 2022), and humans, where PNNs are concentrated in layer 3 (Hausen et al., 1996). This difference in PNN layer-specificity raises the question of how PNNs might affect neural input integration, synaptic organization, and overall circuit architecture in the degu brain. Previous studies identified layer-specific inputs to S1, with middle layers receiving thalamic projections and superficial/deeper layers receiving more cortical inputs, such as from the primary motor cortex (Yu et al., 2019; Zhang and Bruno, 2019). Further, as deep cortical layers function as prominent output regions of the cortex, this suggests PNNs trigger circuit maturation in areas involved in both input processing and output modulation (Thomson, 2010; Moberg and Takahashi, 2022). As PNNs ensheath of variety of neuronal subtypes, with the most prominent being GABAergic interneurons, a full characterization of the PNN-colocalized subtype distribution in a layer-specific manner would provide crucial insight on how PNNs might be modulating local cortical circuitry in the degu brain (Oohashi et al., 2015; Fawcett et al., 2019). Although projection patterns and neuronal subtype cytoarchitecture need to be explored, it would be interesting to investigate why deep layer neurons in the degu show such dense PNN enwrapping.

We find degus also possess a novel PNN expression pattern in the dorsal hippocampus, characterized by robust signal in hippocampal CA3 (highest in CA3a), dentate gyrus, and minimal-to-no detectable PNN signal in CA1 (Figure 6). EC and SUB also exhibit prominent PNNs, revealing CA1 shows the least PNN signal in the hippocampal formation. This pattern of PNN expression differs from those seen in other rodents. Mice PNNs are present throughout their hippocampus proper. Rats primarily express PNNs in CA2 and CA3b, suggesting rats have a more restrictive PNN expression pattern along the CA1-to-DG axis (Lensjø et al., 2017). Humans, on the other hand, express PNNs in all subfields of the hippocampus, with the greatest amount occurring in their hippocampal CA1 stratum oriens (Lendvai et al., 2013). As the hippocampus possesses major roles in learning, memory, and spatial navigation (Andersen et al., 2006), these differing PNN expression patterns could reflect species and region-specific functional plasticity requirements. Further studies looking at how differential regional and laminar PNN expression patterns correlate with differing neural circuitry, synaptic architecture, and behavior could provide important insights on the range of species-specific matrisome profiles seen in the mammalian brain.

Although Iba1 relative intensity was not statistically different between the younger and older adult age groups, we observed greater Iba1 relative intensity variance in the older adults in 8 of the 9 analyzed brain regions. Further, older adult degu Iba1 intensity means were higher than those of the younger adults in 7 of the 9 analyzed brain regions (Table 5). This increased variance and average iba1 intensity values could suggest a portion of the older adult degu population is manifesting divergent microglial states. As degus live up to ~8 years in captivity, it would be interesting to see if this subtle change occurring in 2–3-year-old degus could foreshadow glial and neural changes that develop in the aging degu. Previous studies identified amoeboid microglia morphologies in 5-year-old degus presenting Alzheimer's disease-like pathology (Tan et al., 2022), while our current study mostly identified healthy ramified microglia morphologies (Streit et al., 2014). Future studies looking into how degu microglial populations progress in healthy and diseased elder age will help clarify if diversified microglial expression patterns are occurring in the aging degu.

**Table 5:** Microglia Iba1 relative intensity data summary across degu age groups and brain regions.

**Iba1 relative intensity**					
**Brain region**	**Measure**	**Juvenile (1–3 m.o.)**	**Adolescent (5–8 m.o.)**	**Younger Adult (12–19 m.o.)**	**Older adult (23–40 m.o.)**
PrL	Mean	16.01	15.35	12.92	10.64
	Standard deviation	7.212	4.297	5.853	5.403
EC	Mean	11.73	10.05	7.601	8.113
	Standard deviation	3.62	3.843	3.63	3.883
RSC	Mean	9.558	8.699	4.367	5.85
	Standard deviation	2.509	1.996	0.9597	1.777
TRN	Mean	8.534	5.585	2.385	4.104
	Standard deviation	2.936	3.368	1.575	1.628
CA3a	Mean	15.91	9.903	3.370	7.914
	Standard deviation	3.969	3.465	2.612	5.790
BLA	Mean	20.96	16.71	5.722	13.24
	Standard deviation	6.544	5.465	2.463	4.186
S1	Mean	28.99	17.61	14.91	13.30
	Standard deviation	6.457	4.803	4.828	5.685
SUB	Mean	40.64	16.77	7.868	19.92
	Standard deviation	9.498	8.639	4.619	9.987
CA1	Mean	13.95	10.95	4.374	11.21
	Standard deviation	3.129	3.754	1.611	6.428

Summary of Iba1 relative intensity means and standard deviations across brain regions in the 4 studied degu age groups (juveniles, adolescents, younger adults, and older adults).

In summary, our results provide a broad illustration of neuroplasticity across the degu lifespan via two mediators of plasticity: microglia and PNNs. We confirm degus exhibit established patterns of mammalian development, with juvenile subjects exhibiting enhanced plasticity states that subside in adulthood. We identify adolescence as a life stage with unique neuroplasticity characteristics, defined by adult-like PNNs coupled with intermediate levels of microglia. We overall find three distinct age-related neuroplasticity states illustrating the forebrain's transition to adulthood: pre-pubescence, adolescence, and young adulthood. Our characterization of PNNs in the degu brain reveals degu somatosensory cortex and dorsal hippocampus have patterns of PNN expression that differ from what is seen in other rodents and humans. Taken together, these results begin to elucidate the neurodevelopmental characteristics of the *O. degus*, an animal model gaining traction in social, developmental learning, and age-related neuropathology research. We foresee our results elucidating the maturation of the degu forebrain will help build a foundation for a broader comparative understanding of neural and cognitive development.

## Data availability statement

The original contributions presented in the study are included in the article/supplementary material, further inquiries can be directed to the corresponding authors.

## Ethics statement

The animal study was approved by University of Montana's Institutional Animal Care and Use Committees and the Institute of Ecology and Biodiversity Ethics Committee, University of Chile, Santiago, Chile. The study was conducted in accordance with the local legislation and institutional requirements.

## Author contributions

BG: Conceptualization, Data curation, Formal analysis, Investigation, Methodology, Visualization, Writing—original draft, Writing—review & editing. PH: Data curation, Investigation, Writing—review & editing. CH: Formal analysis, Investigation, Writing—review & editing. PC: Resources, Writing—review & editing. NI: Conceptualization, Methodology, Resources, Writing—original draft, Writing—review & editing. XX: Conceptualization, Funding acquisition, Methodology, Project administration, Resources, Supervision, Writing—review & editing.
